# Commentary: “Physical time within human time” and “Bridging the neuroscience and physics of time”

**DOI:** 10.3389/fpsyg.2023.1087695

**Published:** 2023-06-14

**Authors:** Nick Huggett

**Affiliations:** Department of Philosophy, University of Illinois Chicago, Chicago, IL, United States

**Keywords:** time perception, illusion, motion perception, philosophy, flow of time

## 1. Introduction

Consider Zeno's of Elea's paradox of the arrow, propounded in the fifth century BCE: when does the arrow move between points *p* and *q*, given (a) that instants of time are indivisible? Not *during* any instant, since then it would be divisible into an earlier part (when the arrow is at *p*) and a later part (when it is at *q*). If not during, then it could only be *between* instants; but given (b) that time is completely composed of instants, between them is no time at all. In other words, the arrow is stationary, moving at *no* time, contrary to experience!

A delightful argument, which cuts to the heart of the question of what a mathematical function is, something that was not fully understood until the nineteenth century development of analysis (Huggett, 2019). That conception, of course, is that functions—say, the position of the arrow—are *not* objects that “flow” or “move” with their arguments in some primitive, intuitive, and even experiential (but ultimately unexplicated) sense, as Zeno seems to assume (to demonstrate their non-existence). Instead, they are simply a pairing of each argument-value to a unique function-value, and concepts such as motion, flow, or continuity are then *defined* within this picture, using the limit concept of Bolzano, Cauchy, and Weierstrass (cf Courant et al., 1996, §V.3). For motion, we then have the “at–at” theory—the motion of the arrow entirely consists of its being *at* a place *at* each time,—while all that remains of flow is the differentiability of the series of places with respect to the series of times.

I start with this familiar example to illustrate the problems which the two manifestos, Buonomano and Rovelli (2021) (henceforth B&R) and Gruber et al. (2022) (GBM) seek to address. That is, there is a gap—or better, gaps, since the articles correctly emphasize time's multifacetedness—between the “everyday” conception of time, and the scientific (specifically physics') conception, and between the philosophical elaborations of those concepts. Similar to GBM, let us refer to the former concept in Willfrid Sellar's terms as “manifest.”^1^ As the articles note, recognition of such gaps is as old as philosophy, and indeed one could read Zeno in this way: “manifestly the arrow moves, but by the lights of the contemporary physics of time circa 450BCE—including assumptions (a) and (b), and a view of functions as flowing—it cannot.” As we just saw, Zeno's argument was undermined by (ironically!) changes in the scientific conception of time and change, specifically the development of analysis. However, that very development opens a new gap: some supposed experiential idea of temporal “flow” in motion on one side, which is rejected for the at-at picture of time and change on the other.

This idea of flow is arguably unexplicated beyond metaphor by its proponents, and I have no precisification to offer; except in the sense that I will argue that what people call “flow” is in fact something quite different. Under the circumstances, I hope that the vagueness in the term will be excused [NB: my target is motion as flow, which I will generally refer to as temporal flow, even though that arguably has other facets (Callender, 2017, chapter 11)].

Both B&R and GBM argue for a two-pronged attack on the general problem of the physical-manifest gap: on the physical side (including the physiological aspect of neuroscience), explain the physical environment and mechanisms underwriting temporal experience; on the psychological side, classify and explain the veridical and non-veridical experiences that result. As the references to the articles illustrate, many philosophers of physics, including myself, have taken this approach and I am very congenial to these arguments (and welcome increased interaction with psychologists). The alternatives seem to be either to abandon the idea of the unity of science, or radically rethink physics; neither option seems palatable (I argue against the latter in Huggett (2014), which also discusses the following example).

My aim here, then, is to pursue such an approach to “flow,” but thereby offer an important friendly clarification of that approach. In particular, “illusion” is defined and used too loosely, obscuring some distinctions that are important for the explanation of temporal experience: “illusion refers to a perception that has no basis in reality” (GBM, p. 3).^2^ Trivially, all perceptions have some “basis” in reality since they are caused by something real, so GBM has something more restrictive in mind: that there is some special relation *X* in which veridical perceptions stand with respect to the world; for instance, that they properly *represent* their objects, while illusions do not. Now, the question of perceptual content is an entire sub-field of philosophy, which I cannot settle here. However, my argument is largely independent of any specific account: all that matters is that some relation between percept and world (“basis in reality”) holds for veridical perceptions, and fails to hold for illusions. Let us call that relation “representation,” but without overburdening it with philosophical baggage.

Then my clarification will be that there can be veridical perceptions, properly representing physical time, which nonetheless lead to a manifest conception of time at odds with the scientific conception. For GBM, it seems that only an illusion could lead to an erroneous conception of time, whereas I will claim that it is also possible to be *mistaken* about what it is that a veridical perception represents. For instance, in the waterfall illusion, one has a percept of motion, where there is none. But imagine that on seeing a stranger in the street, I mistake them for a friend. But did I suffer an illusion, and perceive someone who was not there? Perhaps instead the perception properly represents the stranger, but I am mistaken about who it represents. In the following, I propose an analogous analysis of the supposed perception of flow.

## 2. “Illusions” are not what they seem

Consider the well-established motion detection mechanism, a Reichardt detector, thought to be implemented in the visual cortex (Mikami et al., 1986).^3^ Crudely, a pair of spatially separated high-contrast edge detectors in the retina (or perhaps lateral geniculate nucleus) are connected, as it were, to a logical AND gate, with a time delay in one input: if a light patch moves across the retina, the first one then the other detector will fire, and if the first signal is delayed for exactly the time it takes the patch to move between the detectors, then the AND gate will fire. Thus, the whole mechanism is a simple detector for edges moving across the retina, and so for the motion of physical bodies; likely one among a variety of motion detectors, for which it will serve as a representative in what follows.

The phenomenon of apparent motion indicates that the outputs of motion detectors enter consciousness, though how is not settled. When they do, the resulting percept amounts to (invisible) mental vector arrows attached to objects in the visual field, indicating their speed and direction of motion; and the perception is veridical to the extent that it properly represents the motion, so to the extent that bodies move as perceived.

Consider the phenomenology of this percept, to see that it is the source of the manifest conception of temporal/motion flow. Motion illusions, including the waterfall and apparent motion can make us quite strongly aware of the percept, but its absence can also be striking. Stroboscopic light below 50 Hz can make moving objects appear to jump from place to place: just what is missing is, I claim, that percept which the folk refers to as “flow.” It is not hard to see how such lighting can thwart motion detection: for instance, a moving object illuminated when it triggers one of the edge detectors in a Reichert detector, may be in darkness when it would otherwise trigger the other, so that motion is not detected—thus, the corresponding motion percept is absent.

Analogously, but far more dramatically, Zihl et al. (1983) famously reports a study of a patient with damage to the visual cortex, specifically around the MT region known to be associated with motion detection. Remarkably the only significant impairment that they suffered was to motion perception but despite the fact that they perceived objects at sequential locations, they (i) reported lacking a motion percept, and (ii) were unable to perform tasks requiring information about motion: “She had difficulty …pouring tea or coffee into a cup because [i] the fluid appeared to be frozen, like a glacier. In addition, [ii] she could not stop pouring at the right time since she was unable to perceive^4^ the movement in the cup …when the fluid rose” (p. 315). The patient reported people and vehicles “suddenly” being “here or there,” without having “seen them moving.” Plausibly, these experiences arose from the integration of static and motion aspects of experience: current motion perception produces expectations of future spatial arrangements of objects, which were continually thwarted by moving objects. Whether objects stroboscopically jumped across her visual field, or moved continuously but to the “wrong” places (supposing such a distinction can be drawn) is unclear from the published reports. Regardless, the reasonable interpretation is that because the patient was lacking normal motion detectors, they were also lacking a characteristic motion percept.

These two pathological cases highlight—by their absence—a component of ordinary experience about which people are often confused. Specifically, motion perception is not merely a matter of experiencing an object in sequential locations, but also an awareness of instantaneous velocity, the “mental vectors” attached to bodies in the visual field. No doubt the reader has also noticed the connection to Zeno's arrow: the gap between the conception of motion in a flowing time and the at-at account of motion parallels the gap between the experiences of a moving body with and without functioning motion detection. Indeed, I submit that *the manifest image of (the motive aspect of) temporal flow ultimately refers to the very percept missing in these two cases* (and not to the visual experience of sequential location that remains): exactly what the folk would say about them is that they involve no experience of motion as flow.

But the neuroscience described indicates that in non-pathological cases this percept represents the at-at motion of bodies; mechanisms like the Reichardt detector work reliably on the basis of physical objects (and hence, the light they reflect) being at sequential locations at sequential times. No kind of “flow” is implicated at all in their proper operation. Moreover, the information that they provide for action is also of the at-at kind: at what place will an object be at a later time? In that case, the corresponding experience represents the at-at motion of the object, and *if one thinks that it is an experience of temporal/motion flow, one is simply mistaken about what it represents*.

However, it cannot quite be right to say that motion percepts are mistaken for flow percepts, since that suggests that there is such thing as a “flow percept.” Given the dubious coherence and arguable non-existence of “flow,” what could a percept of it possibly be? (and if it is nothing, then certainly flow is not an *illusion*, in the sense of having a flow percept without flow in reality). Thus, we should more carefully say that people are mistaken about *the physical correlate of the motion percept*, and the resulting confusion leads to vacuous talk of “flow.”

## 3. Discussion

How then, do these considerations bear on GBM? In their scheme, it seems that the unphysical flow of time must be understood as arising from a “gadget” producing an illusory perception. We have indeed identified a gadget responsible for the concept of time flowing, namely Reichardt detectors and the like. But calling flow illusory erases an important distinction between what is going on here and in other cases: the motion detected and perceived is absolutely real (in the at-at sense), one simply misinterprets it. Nor can this error be understood as a “cognitive add-on” to perception (p3) since it does not modify perception, but misconstrues it.

I then have two programmatic concerns about the veridical/illusion dichotomy in GBM. First, if one had in mind that the supposed perception of flow had to be an illusion, then the desired explanation seems to be unavailable since the motion perceived is real, not illusory. Second, if “illusion” is ambiguous, then so is the dualistic hypothesis; how exactly are we to say whether a component of perception is veridical or illusory? On which side do motion detection and its misinterpretation fall?

## Author contributions

The author confirms being the sole contributor of this work and has approved it for publication.

## Footnotes

^1^In the philosophical literature, it is sometimes also called the “folk” concept: e.g., Latham et al. (2020). This work empirically investigates the question of what gaps between manifest and scientific images of time truly exist. This is important work: too often gaps are posited without careful scientific investigation of what the folk really think. What, for instance, is the folk understanding of temporal “flow,” blithely assumed here, and in the articles? There is more to learn.

^2^GBM acknowledges stretching the concept but, as I will explain, in a different direction from me. The use of, and complaints about, this terminology in the philosophy of time are of course not new: for recent examples see Paul (2010) and Callender (2017, §11.4.1), respectively. For an entry into the large literature in the philosophy of perception concerning the nature of illusion, see e.g., Egan (2014).

^3^An overview of these matters can be found in Mather (2016, Chapter 12).

^4^My footnote: the inability to *perceive* motion does not seem critical; all that matters to explain the failure is the unavailability of the information, whether conscious or not.

## References

[B1] Buonomano D. Rovelli C. (2021). Bridging the neuroscience and physics of time. arXiv [preprint]. 10.48550/arXiv.2110.01976

[B2] Callender C. (2017). What Makes Time Special? Oxford: Oxford University Press. 10.1093/oso/9780198797302.001.0001

[B3] Courant H. R. R. Courant R. Robbins H. Stewart I.. (1996). What is Mathematics?: An Elementary Approach to Ideas and Methods. New York, NY: Oxford University Press.

[B4] Egan A. (2014). “Projectivism without error,” in Perceiving the World, ed B. Nanay (Oxford: Oxford University Press), 68–96. 10.1093/acprof:oso/9780195386196.003.0005

[B5] Gruber R. P. Block R. A. Montemayor C. (2022). Physical time within human time. Front. Psychol. 13, 718505. 10.3389/fpsyg.2022.718505 35432085 PMC9005802

[B6] Huggett N. (2019). “Zeno's paradoxes,” in The Stanford Encyclopedia of Philosophy. Metaphysics Research Lab, ed E. N. Zalta (Stanford, CA: Stanford University), Winter 2019 edition. Available online at: https://plato.stanford.edu/archives/win2019/entries/paradox-zeno/

[B7] Huggett N. (2014). Skeptical notes on a physics of passage. Ann. N. Y. Acad. Sci. 1326, 9–17. 10.1111/nyas.12514 25183288

[B8] Latham A. J. Miller K. Norton J. (2020). Do the folk represent time as essentially dynamical? Inquiry. 10.1080/0020174X.2020.1827027

[B9] Mather G. (2016). Foundations of Sensation and Perception. London: Psychology Press. 10.4324/9781315672236

[B10] Mikami A. Newsome W. T. Wurtz R. H. (1986). Motion selectivity in macaque visual cortex. ii. spatiotemporal range of directional interactions in mt and v1. J. Neurophysiol. 55, 1328–1339. 10.1152/jn.1986.55.6.1328 3734858

[B11] Paul L. A. (2010). Temporal experience. J. Philos. 107, 333–359. 10.5840/jphil2010107727

[B12] Zihl J. Von Cramon D. Mai N. (1983). Selective disturbance of movement vision after bilateral brain damage. Brain 106, 313–340. 10.1093/brain/106.2.313 6850272

